# PiRNA Obtained through Liquid Biopsy as a Possible Cancer Biomarker

**DOI:** 10.3390/diagnostics13111895

**Published:** 2023-05-29

**Authors:** Piotr Limanówka, Błażej Ochman, Elżbieta Świętochowska

**Affiliations:** Department of Medical and Molecular Biology, Faculty of Medical Sciences in Zabrze, Medical University of Silesia, 19 Jordana, 41-800 Zabrze, Poland; s74817@365.sum.edu.pl (B.O.); eswietochowska@sum.edu.pl (E.Ś.)

**Keywords:** piRNAs, cancer biomarker, liquid biopsy, sncRNA

## Abstract

In recent years PIWI-interacting RNAs (piRNAs) have gained the interest of scientists, mainly because of their possible implications in cancer. Many kinds of research showed how their expression can be linked to malignant diseases. However, most of them evaluated the expression of piRNAs in tumor tissues. It was shown how these non-coding RNAs can interfere with many signaling pathways involved in the regulation of proliferation or apoptosis. A comparison of piRNA expression in tumor tissue and adjacent healthy tissues has demonstrated they can be used as biomarkers. However, this way of obtaining samples has a significant drawback, which is the invasiveness of such a procedure. Liquid biopsy is an alternative for acquiring biological material with little to no harm to a patient. Several different piRNAs in various types of cancer were shown to be expressed in bodily fluids such as blood or urine. Furthermore, their expression significantly differed between cancer patients and healthy individuals. Hence, this review aimed to assess the possible use of liquid biopsy for cancer diagnosis with piRNAs as biomarkers.

## 1. Introduction

PIWI-interacting RNAs (piRNAs) are a group of non-coding RNA that can bind to PIWI proteins. piRNAs are single-stranded oligonucleotides built from approximately 26–31 nucleotides and were first described in 2006 [1,2]. One of the first functions of piRNAs that was described was their ability to control transposable elements [3,4,5]. Other significant functions are gene regulation, protein regulation, genome rearrangement, and spermatogenesis [6]. piRNAs are transcripted from clusters located at specific loci [1,3]. De novo transcription of these clusters of piRNAs is conducted by RNA polymerase II [7]. Transcripted piRNA precursors are then cleaved on the 5′ end by Zucchini endonuclease, which results in uridine being nucleotide on 5′ termini [8,9]. Additionally, it is suggested that Zucchini is involved in the formation of a 3′ end [10], and 3′ termini are also 2-O-methylated by HEN1 methyltransferase [11]. The secondary amplification cycle is also called the “ping-pong cycle” and takes place in cytoplasm [3]. Aub and Ago3 proteins are recruited to the subcellular compartment called Nuage. These two proteins are involved in the ping-pong cycle where cleavage products of each protein are passed to the other one resulting in the production of more piRNAs [12,13]. Similar to other small RNAs, the appropriate level of piRNA depend on the balance between their biogenesis and degradation processes. The mechanisms underlying piRNA degradation differ from those of other small RNAs and have not been fully elucidated. PiRNAs appear to be more stable and resistant to degradation and oxidation processes than other small RNAs, such as microRNAs, the role and diagnostic significance of which have been extensively investigated in recent years [14,15,16]. The most important processes that protect piRNA from various degradation mechanisms are 3′ terminal 2′-O-methylation and 3′-to-5′ trimming of piRNA [14,17,18]. The turnover of small RNAs, including piRNAs from their 3′ end, is related to the activity of the 3′–5′ exoribonuclease [19,20]. However, the activator of the decay pathway dependent on the appropriate 3′–5′ exoribonuclease is the earlier uridylation at the 3′ end of the RNA [21,22]. Unlike other human small RNAs, piRNAs contain the aforementioned 2′-O-methyl group at the 3′-end, which protects them against 3′ uridylation and makes them more resistant to degradation [23,24]. The sensitivity of microRNA and piRNA to complementary RNAs triggering degradation differs in their sensitivity to 2′-O-methylation. Animal microRNAs are unmethylated, and miRNAs with extensively complementary targets are unstable. This phenomenon is called target-directed miRNA degradation (TDMD) and is insensitive to 2′-O-methylation. Degradation of unmethylated piRNAs, however, is distinct from TDMD, and 2′-O-methylation inhibits complementarity-dependent piRNA destabilization and reduces its degradation through the exosome-mediated decay pathway [25,26,27]. On the other hand, it has been revealed that the major mechanism for piRNA decay is mediated by the 5′–3′ exoribonucleases XRN1 and XRN2, although the binding of PIWI proteins protects piRNAs from this decay pathway [24,28,29]. These characteristics of piRNA structure and its components appear to contribute to enhanced stability and resistance of piRNA, compared to other small RNAs against degradation processes, both within intracellular and extracellular environments.

The functions of piRNAs have been first examined in germline cells, but since then it was proved that its expression was dysregulated in cancer cells [1,30,31]. It was shown that their expression might be associated with carcinogenesis through different mechanisms. Inhibition of piR-823 resulted in suppressed cell proliferation, arrested the cell cycle in the G1 phase, and induced cell apoptosis in colorectal cancer (CRC) cell lines. Additionally, this inhibition resulted in the repressed expression of heat shock protein (HSP) HSP-27, HSP-60, HSP-70. Overexpression of this piRNA in normal colonic epithelial cell line FHC promoted their proliferation. It was demonstrated that piR-823 binds to heat shock transcription factor 1 (HSF1) and promoted its phosphorylation at Ser326, which resulted in increased transcriptional activity of HSF1 [32]. In cervical cancer cells, piRNA-14633 was shown to promote malignancy through the METTL14/CYP1B1 pathway [33]. piR-021285 was suggested to be linked with the invasiveness of breast cancer through 5′ UTR/first exon methylation of the ARHGAP11A gene. Additionally, it was revealed that SNP rs1326306 G > T in piR-021285 increased the likelihood of breast cancer (BC) [34]. Expression of piRNAs was also altered between heart failure and healthy patients, with has-piR-020009 and has-piR-006426 being the most downregulated [35]. Cancer-altered cells are usually obtained through surgery or biopsy. These methods can be used when a tumor is formed, which means the advancing of carcinoma and delaying diagnosis. Early detection of neoplasm significantly lowers mortality; thus, it is important to develop tools that will enable the detection of cancer as early as possible [36]. PiRNA has been detected in many body fluids, often with a significant change in concentration depending on the disease type and its activity [37]. Extracellular vesicles (EVs) may be responsible for transporting RNA, including piRNA, into body fluids. Depending on their subcellular origin, EVs differ in size as well as the cargo they carry [38,39]. By transferring its cargo (proteins, RNAs, lipids, and the other molecules), EVs are involved in cancer progression and metastasis. They can also influence the invasiveness of cancer cells and participate in the degradation of the extracellular matrix [40,41,42]. The presence of piRNAs has been demonstrated in EVs found in body fluids; however, expression levels of piRNA relative to other small RNAs can vary widely depending on the type of examined body fluid and examined comorbidities [37,43,44]. It was also suggested that piRNAs, because of their relatively short length, can pass through cell membrane. This combined with their stability allows for their detection in body fluids [45].

Liquid biopsy is the method of obtaining body fluids such as blood, urine, or saliva with minimal invasiveness. Three main components of liquid biopsy that can be quantified are considered to be circulating tumor cells (CTCs), exosomes, circulating tumor DNA (ctDNA), miRNA, and piRNA [46,47]. This method is considered to be a promising way of detecting cancer mainly because of its simplicity, non-invasiveness, and low cost [48]. In this review, we would discuss the research progress and clinical utility of piRNAs obtained by liquid biopsy in the cancer detection, or progression monitoring, and potential further research directions devoted to piRNAs in the context of cancer diagnostic biomarkers. This review is based on the articles searched using PubMed, encompassing the literature published over the last twelve years (2011–2023) in cases investigating the diagnostic role of proper piRNAs. However, in the other cases cited from the literature, older articles are also cited. The eligibility criteria were as follows: in vivo studies; studies performed on humans; and studies that resulted in significantly dysregulated expression of piRNA obtained through liquid biopsy in serum, saliva, urine, and gastric juice.

## 2. Colorectal Cancer

In 2020, the incidence of CRC was 1.9 million and is predicted to reach 3.2 million cases in 2040 [49,50]. Widely known methods for diagnosis of CRC are colonoscopy or fecal occult blood test. There are a few new emerging biomarkers, which include proteins, DNA, RNA, low molecular weight metabolites, or shifts in gut microbiome composition [51]. piRNAs are a group of ncRNA with growing interest that can be found in the blood of patients with CRC.

Expression of piR-5937 was altered in non-cancer diseases. Its expression was downregulated in various stages of liver disease that leads to hepatocellular carcinoma [52]. This suggests involvement of piR-5937 not only in cancer but also other diseases, and such a connection needs to be further examined. Similarly exosomes from asthenozoospermia patients had a decreased expression of piR-5937 in comparison to normozoospermic men [53], whereas, piR-28876 was described only in terms of CRC.

Both piR-5937 and piR-28876 were found to be significantly downregulated in patients with CRC. Samples were collected from 403 colon cancer patients and 276 healthy donors. The area under the curve (AUC) of piR-5937 and piR-28876 was respectively 0.7673 and 0.7074. However, the combined results of these ncRNAs did not show an increase of AUC, supposedly because of the correlation between these two. In comparison with CEA and CA19-9, which are known biomarkers for CRC, both piR-5937 and piR-28876 showed better sensitivity, but still, the best result was achieved by combining all four of them. This study showed how mentioned oligonucleotides can be biomarkers for CRC, but it is worth mentioning that expression profiling was performed on pooled samples [54]. Additionally, during diagnosis of CRC, it is necessary to take into consideration that patients with asthenozoospermia also had downregulated expression of piR-5937.

Mai et al. revealed upregulated expression of piR-54265 in both tumor tissue and serum in a CRC stage-dependent manner. The study included 317 serum samples showing the correlation between progression-free survival (PFS), overall survival times (OS), and expression of piR-54265. Furthermore, it was reported that this ncRNA has an association between its expression and curative effect of treatment of 5-FU and oxaliplatin with AUC being 0.811, sensitivity of 66.7%, and specificity of 88.5% of accurately distinguishing the disease progressors from non-progressors after neoadjuvant chemotherapy [55]. Two years later authors published the study that focused solely on the expression of piR-54265 in serum of patients with CRC. This time a larger group of people with CRC were examined and their piR-54265 expression was assessed by using droplet digital PCR. Again, it was presented that this piRNA was not only a possible biomarker for the efficacy of chemotherapy but also could distinguish CRC from other carcinomas of digestive system with AUC of 0.946 and CRC from the control group with AUC of 0.896. Three patients showed downregulation of piR-54265 after removal of the tumor and again upregulation after relapse of CRC; however, due to the small size of the study group, studies with a higher number of patients are needed to confirm that piR-54265 can be an indicator of relapse. Additionally, piR-54265 showed better sensitivity than CEA, CA19-9, CA125, and methylated SEPTIN9 [56]. Tosar et al. suggested that the results of these studies were incorrectly interpreted as small nucleolar RNA SNORD57 [57]. However, the authors of mentioned studies proved that it was indeed piR-54265 and not SNORD57 [58].

piR-019825 was found to be the most prevalent piRNA in urine and cell-free urine and second most prevalent in serum [46]. This implies the importance of piR-019825 in organisms. However, it is important that this fact might also mean that piR-019825 is not cancer specific.

The study that profiled circulating extracellular RNA in control patients and individuals with CRC, pancreatic cancer, and prostate cancer showed the presence of piR-019825 in the blood of patients with CRC. This piRNA combined with five other miRNAs could predict CRC with an AUC of 0.81 [59].

Among these five piRNAs, expression of piR-004153 was found to be connected with smoking [60,61], and the level of piR-020365 in EV was associated with shorter survival of glioblastoma patients [62]. It is worth mentioning that smoking is associated with prevalence of CRC [63].

Qu et al. confirmed the presence of five piRNAs (piR-001311, piR-004153, piR-017723, piR-017724, and piR-020365) that were differentially expressed in the serum of patients with CRC and healthy controls. The number of samples was 220 of both CRC patients and healthy controls. It was shown that these piRNAs were downregulated. The authors constructed the piRNA-based panel, which showed an AUC of 0.862 in a five-piRNA panel. Interestingly this panel performed better than the panel formed with CEA and CA19-9 and had significantly higher AUC. It is worth noting that the expression of one of the piRNAs, piR-017724, was an independent prognostic factor for PFS and OS in CRC patients [64].

piR-020450, similar to piR-004153, was previously reported to be associated with smoking [60,61].

Both piR-020619 and piR-020450 were also found to be promising CRC biomarkers. Their combined panel showed good performance in detecting early stage CRC with an AUC of 0.839, which was significantly higher than the AUC of CEA (0.595) and CA19-9 (0.536). The authors also analyzed the expression levels of piR-020619 and piR-020450 in sera of patients with lung, breast, and gastric cancer, which showed no difference in comparison to normal controls. This suggests that they might be CRC-specific. Expression of these two ncRNAs is also downregulated after removal of the tumor, which additionally suggests a connection between their expression and the presence of CRC [65]. Worth mentioning is the observed expression of piR-017723 in this study, which was suggested to be not significantly different between CRC patients and healthy controls. This finding is contradicts that previously mentioned, where piR-017723 was found to be significantly downregulated [64].

It was revealed that piR-823 was not present in the serum of healthy donors, but it is worth noting that three samples were analyzed [66]. Although piR-823 was found in CRC [32,67,68], gastric cancer [69], multiple myeloma [70,71,72], breast cancer [73] and esophageal [74] cancer where it showed different expression in cancer cells, and played a role in multiple mechanisms within these cells, their expression was measured in cancer and adjacent tissue.

However, piR-823 was also found in the serum of individuals with CRC [75,76]. This piRNA level was upregulated in CRC patients’ serum, which also correlated with the stage of the tumor, where its level was significantly higher in individuals with stage III or IV [75]. Another study conducted on serum samples of CRC patients with the use of the photoelectrochemical (PEC) method showed a higher concentration of piR-823 in these subjects. However, it is worth noting that the number of CRC patients and healthy controls enrolled in this study was 15 and 11 respectively [76].

PEC was also used to assess the level of piR-31,413 in diseased CRC patients [77]. In this study, it was also upregulated but again samples of only 13 patients with CRC were used, which suggests the need for such a study with a much larger number of samples.

## 3. Breast Cancer (BC)

In 2020, the most common cancer was BC, representing 11.7% of all cancer cases worldwide [50]. Diagnosis is usually made through breast imagining (mammography and breast ultrasound), and malignancy is verified by core biopsy [78]. Liquid biopsy has qualities that surpass tissue biopsy, as it is easier to perform and allows results to be acquired more often to better monitor the effects of treatment [79].

It was reported that the level of piR-36743 in serum differed in patients with triple-negative breast cancer (TNBC) who achieved complete clinical response (cCR) during neoadjuvant chemotherapy (NACT) and with patients who did not achieve a such response. This difference was assessed before the third cycle of NACT. Samples from eight patients with TNBC were analyzed, which indicates only the preliminary character of these findings. This piRNA was not found in the urine of patients with TNBC. Additionally, piR-36743 and its expression in serum could not distinguish healthy controls from patients with breast cancer. However, its secreted microvesicular level was altered by chemotherapeutic treatment. These findings suggest that piR-36743 might serve in monitoring the effectiveness of treatment but not in early diagnosis of BC. The very small number of samples implies a need for a larger cohort to assess the possible use of piR-36743 [80].

Previously piR-651 was reported to be involved in the pathogenesis of lung cancer [81]. Additionally, piR-651 had an impact on the upregulation of cyclin D1 and CDK4, which could result in tumor formation in non-small cell lung carcinoma [82].

Zhang et al. examined the expression of piR-651 in the plasma of BC patients with the use of a universal catalytic hybridization assembly system (uniCHA). It could distinguish healthy controls from diseased patients with an AUC of 0.9207. This piRNA combined with miR-1246 achieved AUC of 1.000 [83].

Another study shows the expression of piR-651, piR-17458, and piR-20485 to be significantly downregulated in 37 BC patients in comparison to 33 healthy controls [84]. Most of the patients had TNM stage II (67.6%). The downregulation shown in this research contradicts the previously mentioned study where piR-651 was shown to be upregulated [83]. Additionally, some of these ncRNAs showed divergent expression in healthy controls, limiting their possible clinical use [84]. Another factor worth mentioning is the absence of piR-651 in the serum of healthy donors reported by Yang et al. [66].

## 4. Gastric Cancer

Gastric cancer (GC) is the fourth leading cause of death by cancer worldwide with more than 750,000 deaths in 2020 [50]. This carcinoma is rare among people under 45 years of age and is approximately twice as frequent in men as in women [85]. GC is usually diagnosed with the use of endoscopic biopsy as well as endoscopic ultrasound, CT, or PET-CT [86].

piR-651 and piR-823 were used as markers of circulating tumor cells of GC. Interestingly, their expression was lowered in postoperative patients. piR-823 was found to be associated with T stage and distant metastasis; however, only six samples from patients with M1 score were analyzed. piR-651 was differentially expressed between adenocarcinoma and signet ring cell carcinoma, although only nine signet ring cell carcinoma samples were used. The AUC of these piRNAs were 0.841 in terms of piR-651, 0.822 for piR-823, and 0.860 when they were combined. Additionally, these ncRNAs were shown to be more sensitive in the detection of GC than CEA and CA19-9 [87].

Gastric juice is another liquid that can be used to diagnose GC. piR-1245′ expression was found to be upregulated in the gastric juice of patients with GC. The AUC of piR-1245 was 0.8850 and was higher than that of CEA and CA724 which were 0.642 and 0.673, respectively. In addition, characteristics such as TNM stage, tumor size, OS, and PFS were correlated with the level of piR-1245 in gastric juice, which gives this oligonucleotide a possible prognostic value [88].

Serum exosomal level of three piRNAs—piR-019308, piR-004918, and piR-018569—was upregulated in GC patients’ serum. Based on their expression, their AUC was 0.820, 0.754, and 0.732, respectively, which was higher than that of CEA, AFP, and CA19-9. Levels of piR-004918 and piR-019308 were correlated with metastasis, which could serve as a tool for monitoring the metastasis of GC [89].

## 5. Other Types of Cancer

Exosomal piR-hsa-164586 was found to be significantly upregulated in non-small cell lung cancer (NSCLC). Its AUC considering stage I patients was 0.623, and it was higher than cytokeratin-19-fragment (CYFRA21-1), which had an AUC of 0.506. The level of piR-hsa-164586 was significantly lower before surgery than after [90].

Li et al. reported that expression of piR-hsa-26925 and piR-hsa-5444 was significantly upregulated in serum exosomes of lung adenocarcinoma (LUAD) patients compared to healthy controls. TNM scores of I or II were found for 82.9% of patients. AUC of these two piRNAs was 0.751 for piR-hsa-26925 and 0.713 for piR-hsa-5444. The two-piRNA panel with both of the mentioned ncRNAs was created and showed an AUC of 0.833. However, no connection between their expression and the clinical characteristics of patients was found [91].

piR-823 was found to be upregulated in the serum and urine of patients with renal cell carcinoma (RCC). Although statistical significance was found, differentiating between healthy controls and patients with RCC was not satisfactory, with an AUC of 0.626 in regard to serum. Additionally, there was no connection between the expression of this piRNA and the clinical characteristic of RCC patients [92].

Expression of piR-823 in extracellular vehicles was significantly correlated with the clinical stage of multiple myeloma [71].

Zhao et al. showed that mitochondrial piR-34536 and piR-51810 were found in serum, but their expression did not differ between patients with clear cell renal cell carcinoma and patients with other non-malignant diseases [93].

Another study showed that four piRNAs—novel_pir349843, novel_pir382289, novel_pir158533, and hsa_piR_002468—were found upregulated in urinary extracellular vesicles of prostate cancer patients. hsa_piR_002468 had the largest AUC (0.783), and the panel with all four piRNAs had an AUC of 0.853 [94].

Three exosomal piRNAs—hsa-piR-1040, hsa-piR-1089, and hsa-piR-1170—had a higher level in the plasma of patients with neuroblastoma. Additionally, hsa-piR-1089 could differentiate neuroblastoma patients from healthy controls with an AUC of 0.933. Its level also correlated with metastasis and high-risk Children’s Oncology Group (COG) classification [95].

piR-651 was downregulated in the serum of patients with classical Hodgkin lymphoma (cHL). After remission, its expression rose to a level similar to that of healthy patients which shows its connection to cHL [96].

Likewise, piR-651 was also found to be downregulated in RCC patients. Constructed ROC had 77.33% of sensitivity and 72.37% specificity. The grade and stage of RCC were not correlated with the level of this piRNA [97].

Expression of piR-10506469 was higher in exosomes of patients with cholangiocarcinoma (CCA) and gallbladder carcinoma (GBC), while piR-20548188 and piR-14090389 were upregulated only in patients with CCA. piR-20548188 and piR-14090389 were associated with malignant grade increases in CCA and GBC, respectively [98].

The level of piR-hsa-5936 in EV of bladder cancer patients and healthy controls was significantly different. On top of that, piR-hsa-5936 was associated with an upward trend to high risk class. This piRNA was found in the urine of patients with RC; however, its expression was not significantly different between patients with RC and healthy controls [99].

The level of expression of piR-162725 could distinguish pancreatic cancer patients from healthy controls. This piRNA was found to be upregulated in the serum of these subjects. Additionally, the diagnostic performance of CA19-9 could be enhanced by combining it with piR-162725 [100].

All of the described piRNAs has been included in the table, with their suggested clinical implication, regulation of the expression, sample number and type of liquid biopsy (Table 1).

## 6. Methodology and Problems with Quantifying piRNAs

The application of next-generation sequencing (NGS) technology has brought about a significant impact on the detection of small RNAs, offering a multitude of advantages and additional possibilities in the realm of cancer diagnostics, particularly in the study of piRNA expression. NGS allows for the simultaneous examination of numerous targets, ranging from hundreds to millions, rendering it highly efficient and surpassing the capabilities of preceding genomic technologies such as the reverse transcription-polymerase chain reaction (RT-PCR). This advancement facilitates the rapid identification and characterization of molecules at an accelerated pace [101,102]. Traditional molecular assays necessitate multiple tests to examine various mutations, which in turn requires larger quantities of tissue. By contrast, NGS technology overcomes this limitation by enabling the simultaneous interrogation of multiple targets within a single test, thereby reducing the tissue amount requirement and providing results for dozens or hundreds of targets [103,104]. A crucial challenge is to identify all functional ncRNAs, including piRNA encoded in the human genome, for which emerging genomic, epigenomic, and bioinformatic approaches will be instrumental. The identification of functional piRNAs is impeded by several challenges, including the incomplete understanding of functional motifs or domains, low expression levels of piRNAs, and the necessity for improved delineation of their regulatory regions. The interpretation of NGS testing outcomes can be complex due to the identification of numerous variants with varying clinical significance [105]. Currently, there exists a variety of bioinformatic algorithms and tools that enable the identification of potentially functional non-coding RNAs (ncRNAs); nevertheless, the process of identifying and assigning functions to these ncRNAs remains challenging due to their intricate secondary structure. The integration of microarray technology with next-generation sequencing (NGS) has the potential to be utilized in the classification of sample groups, such as tumor tissues, where microarrays can serve as a preliminary analysis tool, while NGS can provide more detailed information regarding genetic alterations and expression patterns. While NGS presents extensive opportunities for DNA and RNA sequencing, it also generates substantial amounts of data, necessitating the utilization of advanced bioinformatics tools for data management, storage, and analysis of sequencing data [46,106]. RT-PCR and NGS are the most popular methods of detecting piRNAs. However, there are also other ways to assess the level of expression of these sncRNAs.

### 6.1. Photoelectrochemical Biosensor

piR-31,143 was detected with the use of a photoelectrochemical (PEC) biosensor based on the MoS2@ReS2/Ti3C2 hybrid. The signal was amplified by a duplex-specific nuclease. This method was able to distinguish CRC patients from healthy controls with 100% specificity and an AUC of 0.942. The limit of detection was 23 aM. Additionally, only 10 μL of serum was used to evaluate the expression of piR-31-143 [77].

Sun et al. also used PEC to measure the level of piR-823 in the serum of CRC patients. In this case, black/red phosphorus heterojunction@Bi2Te3 hybrid was used. The calculated limit of detection was 16 aM. A higher level of piR-823 was found among CRC patients and AUC was 0.927 [76].

### 6.2. Universal Catalytic Hybridization Assembly System

piR-651 was quantified in MCF-7 cell-secreted exosomes with the use of a uniCHA system. Additionally, this system was used to distinguish BC patients from healthy control with the use of 100 μL of serum. Worth noting is the time of incubation, which was 3 h. It is a significant drawback in the possible use of this method as a standard procedure. However, the authors mentioned the modified uniCHA method, where piR-16926 was detected with similar sensitivity after only 0.5h of incubation [83].

### 6.3. Stability of piRNA

Another important factor of a good and reliable biomarker is its stability in different conditions.

Serum with piR-54265 was stored at different temperatures for different times, underwent thawing and freezing, and no significant changes were found [56].

Similarly, serum containing piR-001311, piR-004153, piR-017723, piR-017724, and piR-020365 was incubated for up to 24 h in room temperatures, incubated for up to 3 months at −80 °C, and underwent up to four repetitive freeze-thaw cycles. Even after being exposed to these conditions, no significant differences in expression were found [64].

Aliquots of blood with piR-651 and piR-823 were stored for 0, 1, 3, and 7 days, at a temperature of −20 °C. No significant differences were found in detected levels of these sncRNAs [87].

The serum of LUAD patients was incubated for 0, 4, 8, 12, and 24 h at room temperature, incubated for up to 3 months at −80 °C, and underwent up to eight freeze-thaw cycles. Expression of piR-has-26925 and piR-has-5444 in serum exosomes did not differ significantly after exposure to these factors [68].

However, Kärkkäinen, Heikkinen et al. raised an important question in terms of the methodology of detecting piRNA, suggesting that most of them are mistaken for other ncRNAs [107]. It was reported that most of piRNAs are indeed fragments of other ncRNAs and not strictly piRNA. Similarly Genzor et al. reported the absence of piRNA in COLO205 cells and suggested that most of piRNA are mistaken for other ncRNAs [108].

## 7. Conclusions and Future Perspectives

To summarize, piRNAs can be promising biomarkers for the detection of cancer with the use of body fluids; however, the number of studies, number of samples examined, inconsistencies in them, and questionable results suggest a need for additional, larger studies to draw any certain conclusions. Many studies have shown the good clinical performance of piRNAs in detecting cancer and distinguishing cancer patients from healthy controls. In comparison to other routinely used biomarkers, piRNAs showed higher AUC, which suggests their potential in cancer diagnostic. CEA was suggested to have limited sensitivity, and overall diagnostic performance was not good enough to be used alone, in terms of CRC detection [109]. Similarly, sensitivity and specificity of CA242 and CA19-9 were 0.719 and 0.803, respectively [110]. CEA, CA19-9, and CA72-4 in early stages of cancer have limited sensitivity [111]. These characteristics of most popular biomarkers indicates a need for biomarkers that have higher sensitivity and specificity in early stages of cancer. PiRNAs might be one of the candidates for such markers. Another characteristic of these oligonucleotides is their stability. Even after exposure to rough conditions, their expression in body fluids did not differ significantly [66].

Nevertheless, the utility of piRNAs as diagnostic biomarkers confronts several significant research challenges. In particular, the comparatively low expression levels of piRNAs in body fluids, in contrast to other small RNAs like miRNAs, can impede their detection and analysis. So far, the highest content of piRNAs relative to other small RNAs and to the other body fluids has been demonstrated in plasma, which suggests that plasma may be the optimal material for investigating the diagnostic significance of piRNAs [46]. Compared to the expression of other small RNA molecules, non-serum body fluids exhibited relatively low expression of piRNAs. Particularly low levels of piRNAs were observed in urine, where significant differences in piRNA expression were also observed between genders, significantly influencing the interpretation of potential diagnostic results and requiring additional regulations [37,46]. The lower percentage of piRNAs compared to the content of other small RNAs, including miRNAs, in body fluids may impact the diagnostic value of piRNAs in the analysis of expression in body fluids in the context of neoplastic diseases. The significance of piRNAs as potential cancer biomarkers is based on their specific expression and changes in response to disease development. However, if piRNAs constitute only a small portion of the RNA composition in a given body fluid, it may hinder the detection of piRNA expression changes associated with cancer. This means that the amount of piRNAs available for analysis is limited, which can make it challenging to identify differences in piRNA profiles between samples or to establish the associations of these molecules with specific disease states. On the other hand, piRNAs may potentially exhibit greater stability in body fluids and be more specific to selected cellular processes and events, including those significantly dysregulated during tumorigenesis. The interplay of these relationships and implications, coupled with the progress and improved accessibility of NGS and other analytical techniques, position piRNAs as promising research targets in the quest for diagnostic markers detectable in body fluids. Moreover, variations in piRNA expression levels across different body fluids and in the presence of comorbidities can introduce complexity in interpreting diagnostic results, underscoring the necessity for additional standardization.

Further investigation is warranted to expand our understanding of the involvement of piRNAs in diseases beyond cancer and to elucidate their potential roles in various cellular processes. It was shown how piRNAs can be expressed in different matter in depending on many factors, and it is crucial to determine these factors to be able to optimize the diagnostic abilities of these sncRNAs. Moreover, diversity in progression of cancer is needed to better clarify how piRNAs can be used in early detection of neoplasm, treatment monitoring, or reactions to certain drugs. Furthermore, exploring the correlation between piRNA expression and treatment responses can optimize personalized therapy approaches. To propel the field forward, future research efforts should concentrate on widening studies on piRNA profiles in different cancer types and stages. This concerted effort would enable the establishment of comprehensive diagnostic panels capable of accommodating the heterogeneous landscape of cancer. Additionally, comprehending the underlying mechanisms governing piRNA degradation and investigating their stability in both intracellular and extracellular environments are imperative for ensuring precise and dependable detection methods.

## Figures and Tables

**Table 1:** Expression of piRNA in different cancer types, and their possible clinical implication.

piRNA	Regulation of the Expression	Sample Number	Type of Liquid Biopsy	Suggested Clinical Implication	Reference
	**Colorectal Cancer**				
piR-5937	Downregulated expression	276 healthy donors, 403 CRC cases	Serum	Diagnostic biomarker	Vychytilova-Faltejskova et al., 2018 [54]
piR-28876	Downregulated expression	276 healthy donors, 403 CRC cases	Serum	Diagnostic biomarker	Vychytilova-Faltejskova et al., 2018 [54]
piR-54265	Upregulated expression	317 CRC cases	Serum	Prognostic	Mai et al., 2018 [55]
piR-54265	Upregulated expression	209 healthy controls, 725 CRC cases, 1303 patients with other types of digestive cancer, 192 patients with benign colorectal tumors	Serum	Prognostic and diagnostic biomarker	Mai et al., 2020 [56]
piR-019825	NA	100 CRC cases, 50 healthy controls	Plasma	Diagnostic biomarker	Yuan et al., 2016 [59]
piR-001311	Downregulated expression	220 CRC cases, 220 healthy controls	Serum	Diagnostic biomarker	Qu et al., 2019 [64]
piR-004153	Downregulated expression	220 CRC cases, 220 healthy controls	Serum	Diagnostic biomarker	Qu et al., 2019 [64]
piR-017723	Downregulated expression	220 CRC cases, 220 healthy controls	Serum	Diagnostic biomarker	Qu et al., 2019 [64]
piR-017724	Downregulated expression	220 CRC cases, 220 healthy controls	Serum	Prognostic and diagnostic biomarker	Qu et al., 2019 [64]
piR-020365	Downregulated expression	220 CRC cases, 220 healthy controls	Serum	Diagnostic biomarker	Qu et al., 2019 [64]
piR-020619	Upregulated expression	327 CRC cases, 327 healthy controls, 50 lung cancer cases, 50 breast cancer cases, 50 gastric cancer cases, 40 CRA cases	Serum	Diagnostic biomarker	Wang et al., 2020 [65]
piR-020450	Upregulated expression	327 CRC cases, 327 healthy controls, 50 lung cancer cases, 50 breast cancer cases, 50 gastric cancer cases, 40 CRA cases	Serum	Diagnostic biomarker	Wang et al., 2020 [65]
piR-823	Upregulated expression	84 CRC cases, 75 healthy controls	Serum	Diagnostic biomarker	Sabbah et al., 2021 [75]
piR-823	Upregulated expression	15 CRC cases, 11 healthy controls	Serum	Diagnostic biomarker	Sun et al., 2022 [76]
piR-31,143	Upregulated expression	13 CRC cases, 8 healthy controls	Serum	Diagnostic biomarker	Li et al., 2022 [77]
	**Breast Cancer**				
piR-36743	Upregulated expression	4 patients receiving a cCR during NACT, 4 patients not achieving a cCR during NACT	Serum, Urine (Upregulation was not observed)	Prognostic	Ritter et al., 2020 [80]
piR-651	Upregulated expression	21 BC cases, 13 healthy controls	Plasma	Diagnostic biomarker	Zhang et al., 2022 [83]
piR-651	Downregulated expression	37 BC cases, 33 healthy controls	Plasma	Diagnostic biomarker	Yin et al., 2021 [84]
piR-17458	Downregulated expression	37 BC cases, 33 healthy controls	Plasma	Diagnostic biomarker	Yin et al., 2021 [84]
piR-20485	Downregulated expression	37 BC cases, 33 healthy controls	Plasma	Diagnostic biomarker	Yin et al., 2021 [84]
	**Gastric Cancer**				
piR-651	Downregulated expression	93 GC cases, 32 healthy controls	Blood	Diagnostic biomarker	Cui et al., 2011 [87]
piR-823	Downregulated expression	93 GC cases, 32 healthy controls	Blood	Diagnostic biomarker	Cui et al., 2011 [87]
piR-1245	Upregulated expression	66 GC cases, 66 healthy controls	Gastric juice	Prognostic and diagnostic biomarker	Zhou et al., 2020 [88]
piR-019308	Upregulated expression	70 GC cases, 60 healthy controls	Serum	Prognostic and diagnostic biomarker	Ge et al., 2020 [89]
piR-004918	Upregulated expression	70 GC cases, 60 healthy controls	Serum	Prognostic and diagnostic biomarker	Ge et al., 2020 [89]
piR-018569	Upregulated expression	70 GC cases, 60 healthy controls	Serum	Prognostic and diagnostic biomarker	Ge et al., 2020 [89]
	**Other types of cancer**				
piR-hsa-164586	Upregulated expression	115 NSCLC cases, 47 healthy controls	Serum	Prognostic and diagnostic biomarker	Li et al., 2022 [90]
piR-hsa-5444	Upregulated expression	70 LUAD cases, 57 healthy controls	Serum	Diagnostic biomarker	Li et al., 2021 [91]
piR-hsa-26925	Upregulated expression	70 LUAD cases, 57 healthy controls	Serum	Diagnostic biomarker	Li et al., 2021 [91]
piR-823	Upregulated expression	178 serum samples and 20 urine samples from RCC patients, 101 serum samples and 15 urine samples from healthy controls	Serum, Urine	Diagnostic biomarker	Iliev et al., 2016 [92]
piR-823	Upregulated expression	36 MM patients, 36 healthy controls	Serum	Prognostic and diagnostic biomarker	Li et al., 2019 [71]
piR-51810	No significant differences	30 ccRCC cases, 15 non-malignant disease cases	Serum	-	Zhao et al., 2019 [93]
piR-34536	No significant differences	30 ccRCC cases, 15 non-malignant disease cases	Serum	-	Zhao et al., 2019 [93]
novel_pir349843	Upregulated expression	35 PCa cases, 15 healthy controls	Urine	Diagnostic biomarker	Peng et al., 2021 [94]
novel_pir382289	Upregulated expression	35 PCa cases, 15 healthy controls	Urine	Diagnostic biomarker	Peng et al., 2021 [94]
has_piR_002468	Upregulated expression	35 Pca cases, 15 healthy controls	Urine	Diagnostic biomarker	Peng et al., 2021 [94]
/novel_pir158533	Upregulated expression	35 PCa cases, 15 healthy controls	Urine	Diagnostic biomarker	Peng et al., 2021 [94]
hsa-piR-1040	Upregulated expression	23 NB cases, 7 healthy controls	Plasma	Diagnostic biomarker	Wang et al., 2023 [95]
hsa-piR-1089	Upregulated expression	23 NB cases, 7 healthy controls	Plasma	Diagnostic biomarker	Wang et al., 2023 [95]
hsa-piR-1170	Upregulated expression	23 NB cases, 7 healthy controls	Plasma	Diagnostic biomarker	Wang et al., 2023 [95]
piR-651	Downregulated expression	11 cHL patients at diagnosis and 9 paired samples at complete response, 10 healthy controls	Serum	Diagnostic biomarker	Cordeiro et al., 2016 [96]
piR-651	Downregulated expression	75 RCC patients, 75 healthy donors	Serum	Diagnostic biomarker	Iliev et al., 2015 [97]
piR-14090389	Upregulated expression	45 CCA cases, 29 GBC cases, 55 healthy controls	Plasma	Diagnostic biomarker	Gu et al., 2020 [98]
piR-20548188	Upregulated expression	45 CCA cases, 29 GBC cases, 55 healthy controls	Plasma	Diagnostic biomarker	Gu et al., 2020 [98]
piR-10506469	Upregulated expression	45 CCA cases, 29 GBC cases, 55 healthy controls	Plasma	Diagnostic biomarker	Gu et al., 2020 [98]
piR-5936	Upregulated expression	47 Bladder cancer cases, 46 healthy controls	Plasma, Urine (Upregulation was not observed)	Diagnostic biomarker	Sabo et al., 2020 [99]
piR-162725	Upregulated expression	45 PC cases, 27 healthy controls	Plasma	Diagnostic biomarker	Li et al., 2022 [100]

## Data Availability

Not applicable.
